# Health Beliefs, Diabetes Education, and Physical Activity Engagement Among Persons Living With Diabetes in Ghana

**DOI:** 10.1111/tmi.70208

**Published:** 2026-07-28

**Authors:** Precious Adade Duodu, Joshua Okyere

**Affiliations:** ^1^ Department of Nursing, School of Human and Health Sciences University of Huddersfield Huddersfield UK; ^2^ Department of Nursing, School of Nursing and Midwifery College of Health Sciences, Kwame Nkrumah University of Science and Technology (KNUST) Ghana; ^3^ School of Human and Health Sciences University of Huddersfield Huddersfield UK; ^4^ College of Health Sciences, University of Ghana Accra Ghana

**Keywords:** diabetes, epidemiology, Ghana, physical activity, public health

## Abstract

**Background:**

From the perspective of the health belief model (HBM), persons living with diabetes' (PLWD) perception about the severity of their condition, their perceived susceptibility, perceived benefits, and perceived barriers could determine their participation in physical activity. This study examined physical activity and its associated factors among PLWD in Ghana.

**Methods:**

This cross‐sectional study was conducted in the Ashanti Region of Ghana. The study involved 398 PLWD. A multivariate linear regression assessed the factors associated with physical activity. The results were presented as coefficients (*β*) with the corresponding 95% confidence intervals (CIs). Statistical significance was set at < 0.05.

**Results:**

The majority of participants exhibited moderate levels of physical activity (M = 6.15, SD = 3.99). After adjusting for covariates, higher perceived susceptibility (*β* = 1.27, 95% CI: 0.50, 2.05), perceived benefits (*β* = 1.23, 95% CI: 0.20, 2.27), and perceived barriers (*β* = 0.67, 95% CI: 0.16, 1.17) were independently associated with higher physical activity scores. A higher waist‐to‐hip ratio (WHR) was negatively associated with physical activity (*β* = −5.85, 95% CI: −11.14, −0.56). Compared to males, females had significantly lower physical activity levels (*β* = −0.89, 95% CI: −1.68, −0.11). Being currently in a union (*β* = −3.10, 95% CI: −4.64, −1.57) or previously in a union (*β* = −3.26, 95% CI: −4.92, −1.61) was associated with lower physical activity compared to those who had never married. Regular attendance at diabetes education sessions was strongly associated with higher physical activity (*β* = 3.01, 95% CI: 1.76, 4.27).

**Conclusion:**

Perceived susceptibility, perceived benefits, and perceived barriers emerged as important cognitive determinants of physical activity, underscoring the relevance of health belief constructs in designing interventions. The findings suggest that diabetes management programmes should incorporate behavioural change models and prioritise targeted education and support, particularly for women and those in marital unions, to promote active lifestyles among PLWD in Ghana.

**Abbreviations**
CI: confidence interval
M: mean
PLWD: persons living with diabetes
SD: standard deviation
SDSCA: summary of diabetes self‐care activities
T2DM: Type 2 diabetes mellitus
WHR: waist‐to‐hip ratio

## 1 Introduction

Ghana is experiencing a double burden of disease, where infectious/communicable diseases coexist with chronic/non‐communicable diseases (NCDs) [1]. Consequently, the country has witnessed an increase in NCDs prevalence and mortality [2]. The existing literature identifies type 2 diabetes mellitus (T2DM) as one of the most prevalent NCDs affecting the Ghanaian adult population [3, 4]. It is estimated that 13.1% of the Ghanaian adult population is living with T2DM [4]. Narh et al. [5] also project that the hospitalisation rates for persons living with diabetes (PLWD) will increase by 21.9% between 2022 and 2032. This underscores the public health significance of promoting preventive measures and proper management of T2DM in Ghana.

The extant literature attributes the risk of T2DM to genetics, underlying health conditions, rapid urbanisation and dietary transitions [6, 7, 8]. Additionally, sedentary lifestyles and physical inactivity have been identified as high‐risk factors for T2DM [6, 9]. As such, promoting physical activity has become one of the most common non‐pharmacological interventions to enhance the quality of life of PLWD [10]. There is evidence suggesting that PLWD who engage in physical activities experience an improved quality of life [10], reduced mortality rate by 5.72 folds [11], and better glycaemic control [12, 13].

Despite the aforementioned benefits, engagement in physical activity remains a challenge in many places, including Ghana [14, 15, 16]. For example, Mogre et al. [16] reported that only 26.7% and 33.2% of PLWD participate in at least 30 min of physical activity or in a specific exercise session in Ghana, respectively. One qualitative study [14] conducted in Ghana revealed that issues related to awareness about physical activity, having chronic comorbidities, feelings of embarrassment, and high work demand were major barriers to PLWD's engagement in physical activity. Studies from other jurisdictions have also identified obesity, comorbidities, duration of condition, and low health‐related quality of life as key factors that impede PLWD's engagement in physical activity [17, 18].

We acknowledge that there is some prior evidence on physical activity in relation to T2DM in Ghana [14, 15, 19, 20, 21]. However, these studies have primarily been qualitative [14], focused on the impact of physical activity on quality of life [15], diabetes risk [19], and obesity measured in body mass index [20] or waist‐to‐hip ratio [21]. Moreover, the existing evidence from Ghana [14, 15, 19, 20, 21], as well as other jurisdictions [17, 18], did not consider the health beliefs of PLWD. Theoretically, from the perspective of the health belief model (HBM), the PLWD's perception about the severity of their condition, their perceived susceptibility, perceived benefits, and perceived barriers could determine their participation in physical activity [22]. Therefore, a more comprehensive investigation that integrates these cognitive and behavioural dimensions was warranted to inform effective, context‐specific interventions. This study provides the needed comprehensiveness and bridges the knowledge gap by examining physical activity and its associated factors among PLWD in Ghana.

## 2 Methods

### 2.1 Study Design

A cross‐sectional design was adopted for this study. This design was used to capture a snapshot of engagement in physical activity and its associated factors at a specific point in time, without examining changes over time or establishing causal relationships.

### 2.2 Setting

The study was conducted in the Ashanti Region of Ghana, the second most populous among the country's sixteen administrative regions [23]. The region is home to the Komfo Anokye Teaching Hospital (KATH), a major tertiary referral centre that provides specialised healthcare services not only to the Ashanti Region but also to several adjoining regions, including Bono, Bono East, Savannah, Upper East, North East, Northern and Upper West [24]. Four health facilities with established diabetes clinics were selected as study sites: KATH, Manhyia District Hospital, Ejisu Government Hospital and Juaben Government Hospital (Figure 1). KATH was purposively selected due to its status as a leading tertiary institution. The remaining three district hospitals were randomly selected from a comprehensive list of eligible facilities in the region using Microsoft Excel's random number generator [25]. This random selection approach ensured that each facility had an equal probability of being included in the study.

**FIGURE 1:**
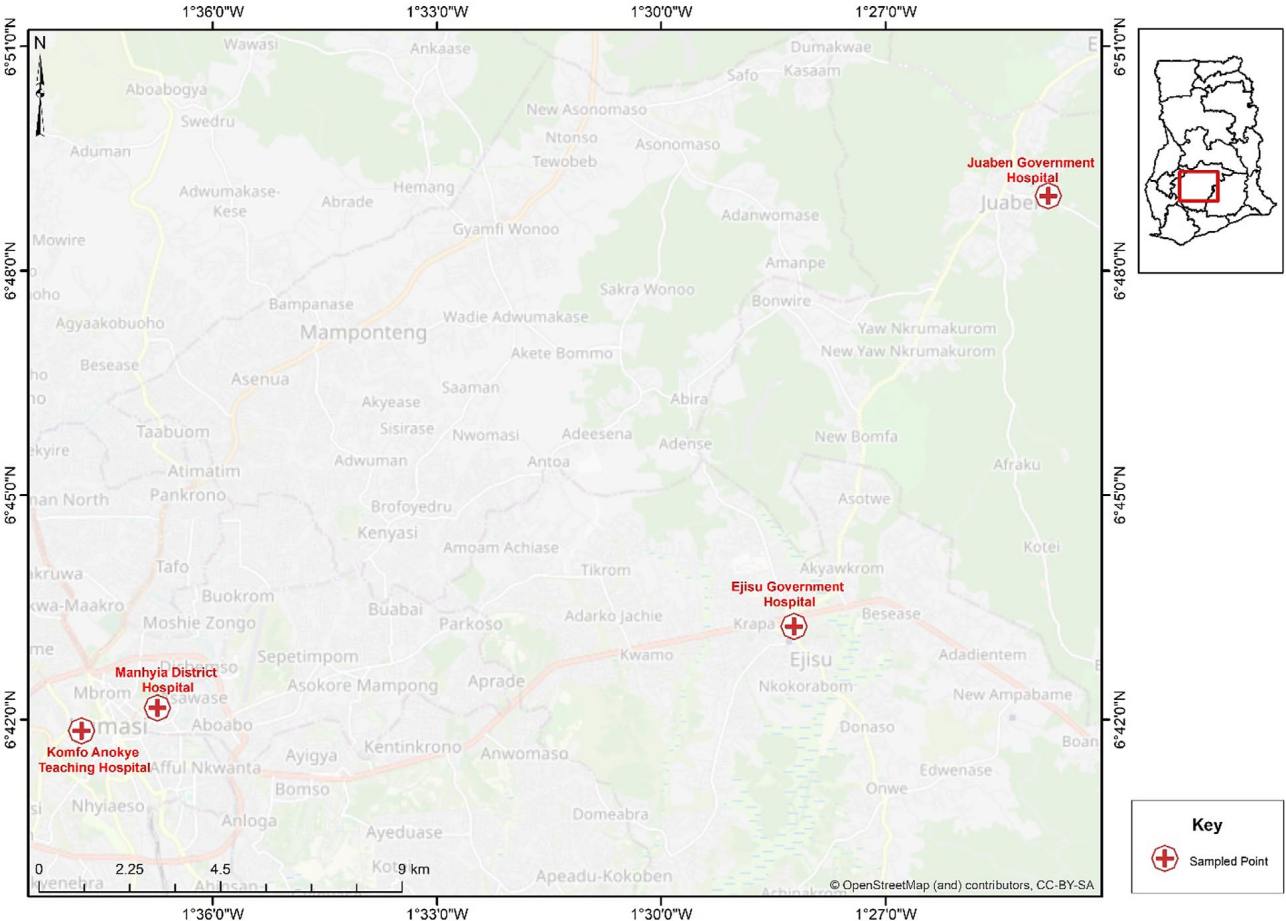
Map of the study sites.

### 2.3 Inclusion and Exclusion Criteria

Participants were eligible to participate only if they were aged 18 years and above with a confirmed diagnosis of T2DM. The disease confirmation must have been based on the World Health Organisation (WHO) diagnostic criteria: a fasting blood glucose level of ≥ 7 mmol/L or a random venous blood glucose level of ≥ 11.1 mmol/L [26]. Eligibility also required that participants had been living with T2DM for at least 6 months before data collection. This minimum duration was intended to ensure that participants had adequate time to adjust to their condition and acquire relevant experience in its management.

### 2.4 Sampling Procedures and Sample Size

The study utilised a convenience sampling approach to recruit participants. Collaboration with administrative staff and healthcare providers at each facility was essential for identifying eligible individuals. Potential participants were screened upon arrival at the outpatient department (OPD), either through a review of registration logs or direct referral by healthcare personnel during the triage process. This approach ensured that all individuals meeting the inclusion criteria were given the opportunity to participate in the study. The sample size was estimated using the formula [27]:

$$\frac{n=z^{2}*p*\left(1-p\right)}{e^{2}}$$

where: *z* = 1.96 for a confidence level (α) of 95%; *p* = projected proportion (50.7%) in Ghana [25]; *e* = margin of error (5%) for greater precision (expressed as a decimal). Thus, *z* = 1.96, *p* = 0.1 and *e* = 0.05. A 5% non‐response rate was calculated on 385, which resulted in a final sample size of 404.

### 2.5 Study Variables

#### 2.5.1 Outcome

The outcome variable was physical activity. This variable was derived from the physical activity component of the Summary of Diabetes Self‐Care Activities (SDSCA) measure. Participants were asked to indicate the number of days they engaged in these two activities in the past 7 days preceding the survey: (a) at least 30 min of physical activity, and (b) a specific exercise session [16, 28]. These two questions were summed to create an index. The Cronbach's alpha coefficient of the SDSCA scale was 0.67 [25].

#### 2.5.2 Explanatory Variables

Previous studies [17, 18] from other contexts have identified sex, age, obesity, comorbidities, and duration of condition as factors that predict PLWD's engagement in physical activities. Also, the general literature on health promotion indicates that an individual's health beliefs could influence their uptake of any health intervention [22, 29]. Based on the reviewed literature, the following were selected as explanatory variables: sex, age, marital status, attending diabetes education sessions, family history of diabetes, having comorbidities, duration of disease, and experiencing chronic complications. Additionally, perceived severity, perceived susceptibility, perceived benefits and perceived barriers (as HBM constructs) were included.

### 2.6 Data Collection Procedures

To support the data collection process, a team of two research assistants and eight data collectors were engaged. These assistants and data collectors were recently graduated nurses who were awaiting placement for their mandatory national service. Prior to data collection, they participated in a three‐day intensive training workshop held 2 weeks before the study commenced. The training took place at Kwame Nkrumah University of Science and Technology (KNUST) and focused on equipping the assistants with a comprehensive understanding of the study objectives, research procedures, and ethical considerations. The sessions included interactive components such as role‐play scenarios, during which the principal investigator offered real‐time guidance and feedback to enhance their readiness. Data collection began immediately after the training and was conducted over a three‐months period, from April to June 2023. Each interview session lasted approximately 45 min [25].

### 2.7 Statistical Analyses

All statistical analyses were performed in STATA version 18 (StataCorp, College Station, TX, USA). For the continuous variables (i.e., physical activity, waist‐to‐hip ratio (WHR), perceived severity, perceived susceptibility, perceived benefits and perceived barriers), means (M), standard deviations (SD) and ranges (minimum [Min] and maximum [Max] values) were computed. For categorical variables such as sex, marital status, attendance at diabetes education sessions, family history of diabetes, duration since diagnosis, comorbidities and chronic complications, absolute frequencies and corresponding percentages were calculated to describe the distribution of participants across each category. To assess the factors associated with physical activity, we performed a multivariate linear regression model. Variables were selected into the model following a backward stepwise approach that iteratively removes statistically non‐significant variables from the model. The coefficients and confidence intervals from the multivariate linear regression were reported. Statistical significance was set at *p* < 0.05.

## 3 Results

A total of 398 participants were included in the analysis (Table 1). The mean physical activity index score was 6.15 (SD = 3.99), with scores ranging from 0 to 14. The average scores for the HBM constructs were as follows: perceived susceptibility (*M* = 4.51, SD = 0.53), perceived severity (*M* = 4.51, SD = 0.52), perceived benefits (*M* = 4.03, SD = 0.56), and perceived barriers (*M* = 2.44, SD = 0.78). The mean waist‐to‐hip ratio (WHR) was 0.92 (SD = 0.08). Most of the participants were females (72.9%), and currently in a union (63.8%). Regarding participation in diabetes education programmes, 60.6% attended sometimes, 18.6% regularly, and 20.9% had never attended. Approximately one‐third (30.9%) reported a family history of diabetes. Most participants (66.3%) had been diagnosed with diabetes for five or more years. Furthermore, 63.6% had comorbid conditions, and only 6.5% reported having any chronic diabetes‐related complications.

**TABLE 1:** Descriptive distribution of the participants' characteristics (*n* = 398).

Variable	*n*	Mean	SD	Min	Max	*n* (%)
Continuous variables						
Physical activity	398	6.15	3.99	0.00	14.00	
Perceived susceptibility	398	4.51	0.53	2.75	5.00	
Perceived severity	398	4.51	0.52	2.75	5.00	
Perceived benefits	398	4.03	0.56	1.60	5.00	
Perceived barriers	398	2.44	0.78	1.00	5.00	
Waist‐to‐hip ratio (WHR)	397	0.92	0.08	0.64	1.36	
Categorical variables						
Age						
Less than 65 years						254 (63.8)
65 years or older						144 (36.2)
Sex						
Male						108 (27.1%)
Female						290 (72.9%)
Marital status						
Never married						12 (3.0%)
Currently in a union						254 (63.8%)
Previously in a union						132 (33.2%)
Attendance to diabetes education						
Never						83 (20.9%)
Sometimes						241 (60.6%)
Regularly						74 (18.6%)
Family history of diabetes						
No						275 (69.1%)
Yes						123 (30.9%)
Years since diagnosis						
Less than 1 year						19 (4.8%)
1 to 4 years						115 (28.9%)
5 or more years						264 (66.3%)
Comorbidities						
No						145 (36.4%)
Yes						253 (63.6%)
Chronic complications						
No						372 (93.5%)
Yes						26 (6.5%)

### 3.1 Physical Activity Distribution Among PLWD in Ghana

Figure 2 presents a kernel density estimate of the physical activity among PLWD in Ghana. The distribution is right‐skewed, with the majority of participants exhibiting moderate levels of physical activity. Also, the highest density was observed between physical activity (PA) index values of 4 and 6, peaking around a value of 5. This suggests that most individuals in the sample engage in moderate physical activity. A small secondary peak was observed around physical activity (PA) index values of 13–14, indicating a minor subgroup of individuals with high physical activity.

**FIGURE 2:**
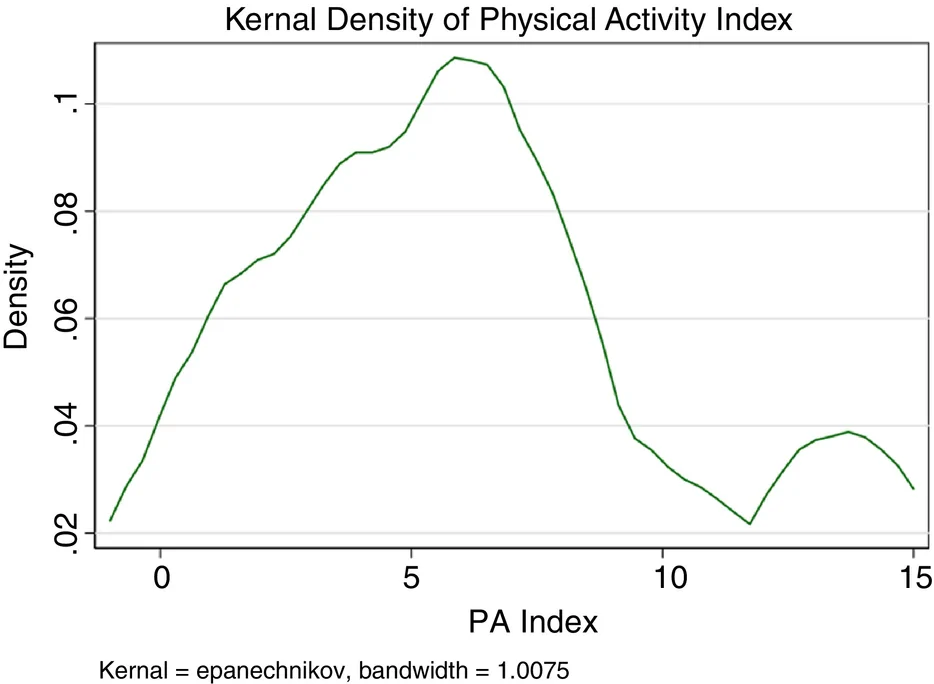
Physical activity distribution among PLWD in Ghana.

### 3.2 Factors Associated With Physical Activity Engagement Among PLWD

Several factors were significantly associated with the physical activity index. After adjusting for covariates, higher perceived susceptibility (*β* = 1.27, 95% CI: 0.50, 2.05), perceived benefits (*β* = 1.23, 95% CI: 0.20, 2.27) and perceived barriers (*β* = 0.67, 95% CI: 0.16, 1.17) were independently associated with higher physical activity scores (see Table 2). A higher waist‐to‐hip ratio was negatively associated with physical activity (*β* = −5.85, 95% CI: −11.14, −0.56). Compared to males, females had significantly lower physical activity levels (*β* = −0.89, 95% CI: −1.68, −0.11). Being currently in a union (*β* = −3.10, 95% CI: −4.64, −1.57) or previously in a union (*β* = −3.26, 95% CI: −4.92, −1.61) was associated with lower physical activity compared to those who had never married. Regular attendance at diabetes education sessions was strongly associated with higher physical activity (*β* = 3.01, 95% CI: 1.76, 4.27). The final model explained approximately 20.6% of the variance in physical activity (*R* ^2^ = 0.2055, *p* < 0.001).

**TABLE 2:** Factors associated with physical activity engagement among PLWD.

Predictor variable	Unadjusted *β* (95% CI)	Adjusted *β* (95% CI)
Perceived susceptibility^a^	1.96 (1.32, 2.60)***	1.27 (0.50, 2.05)***
Perceived severity^a^	1.87 (1.21, 2.52)***	1.23 (0.20, 2.27)*
Perceived benefits^a^	1.98 (1.10, 2.85)***	0.67 (0.16, 1.17)*
Perceived barriers^a^	0.65 (0.12, 1.17)*	−5.85 (−11.14, −0.56)*
Waist‐to‐hip ratio^a^	−3.28 (−8.43, 1.88)	−0.89 (−1.68, −0.11)*
Age		
Less than 65 years	Ref.	—
65 years or older	−0.54 (−1.38, 0.29)	—
Sex		
Male	Ref.	Ref.
Female	−0.35 (−1.18, 0.48)	1.27 (0.50, 2.05)*
Marital status		
Never married	Ref.	Ref.
Currently in a union	−3.28 (−4.98, −1.57)***	−3.10 (−4.64, −1.57)***
Previously in a union	−3.79 (−5.56, −2.02)***	−3.26 (−4.92, −1.61)***
Attendance to diabetes education		
Never	Ref.	Ref.
Sometimes	0.02 (−0.91, 0.96)	0.41 (−0.53, 1.36)
Regularly	2.43 (1.11, 3.76)***	3.01 (1.76, 4.27)***
Family history		
No	Ref.	
Yes	−0.26 (−1.09, 0.58)	—
Years since diagnosis		
Less than 1 year	Ref.	
1–4 years	0.03 (−1.90, 1.97)	—
5 or more years	−0.18 (−2.07, 1.71)	—
Comorbidities		
No	Ref.	—
Yes	−0.56 (−1.46, 0.34)	—
Chronic complications		
No	Ref.	—
Yes	−0.91 (−1.84, 0.02)	—

*Note:* (−) Variables with *p* ≥ 0.05 excluded through a backward stepwise approach. *p < 0.05, **p < 0.01, ***p < 0.001.

^a^ Continuous variables.

## 4 Discussion

Among the recommended non‐pharmacological strategies for managing T2DM, physical activity plays a crucial role in improving glycaemic control, enhancing quality of life and reducing cardiovascular risk factors [10, 11, 12, 13]. This study examined physical activity and its associated factors among PLWD in Ghana. We observed that the majority of PLWD had a moderate engagement in physical activity. This aligns with Volčanšek et al.'s study [12] that reported a moderate physical activity engagement among PLWD. Our finding is corroborated by Amerzadeh et al. [18] whose study reported a moderate level of physical activity among PLWD. The observed moderate physical activity suggests that clinicians and diabetes specialists in Ghana have a lot more to do to encourage PLWD to transition to a high engagement in physical activity. This will be necessary to facilitate maximum protective effects against diabetes‐related complications.

The study revealed that perceived susceptibility was positively associated with physical activity among PLWD. This means that PLWD who consider themselves to be more susceptible to diabetes‐related complications are more likely to engage in physical activity than those with a low perceived susceptibility. Our finding is inconsistent with those of Kim [30] who found an inverse association between perceived susceptibility and physical activity among PLWD in Vietnam. It is also contrary to that of Tamirat et al. [31] who found no statistically significant association between perceived susceptibility and physical activity among PLWD in Ethiopia. Nonetheless, the observed positive association could be explained from the perspective that individuals who recognise their vulnerability to diabetes‐related complications may be more motivated to take preventive actions, such as engaging in physical activity, to mitigate those risks. In the Ghanaian context, where access to healthcare may be limited, and the burden of managing diabetes largely falls on the individual, heightened awareness of personal health risks could serve as a strong internal driver for behaviour change. This sense of susceptibility may enhance health consciousness and prompt proactive efforts to avoid adverse outcomes.

We observed a positive association between perceived severity and PLWD's engagement in physical activity. This aligns with studies conducted in Vietnam [30], Ethiopia [32] and the United States [33]. A plausible explanation could be that perceiving diabetes as a condition with potentially severe consequences, such as vision loss, kidney failure, amputations, or even death, can heighten a sense of urgency and responsibility to take control of one's health. In this case, physical activity may be seen as a concrete and accessible strategy to reduce the risk of complications and improve overall wellbeing. Consistent with existing literature [32, 34], we found that high perceived benefit was associated with high engagement in physical activity. The result can be explained from the perspective that PLWD who have a high perceived benefit are most likely to be aware of the need to stay physically active, as well as all the health benefits that come with being active. Therefore, as rational beings, they will engage in some cost–benefit analysis and try to maximise the benefits that they can accrue from engaging in physical activity.

PLWD who had a high perceived barrier were less likely to engage in physical activity. This finding is consistent with previous studies conducted in Ghana [14], Malawi [35] and the United States [36], which similarly identified perceived barriers as a major determinant of reduced physical activity participation among PLWD. These barriers are often multifaceted, encompassing both psychological and structural challenges. For instance, fear of public ridicule can lead to social discomfort and avoidance of physical activity. Additionally, the presence of comorbidities such as hypertension or musculoskeletal pain may limit physical capacity, leading individuals to perceive exercise as physically demanding or risky. Beyond physical limitations, limited access to safe and appropriate exercise spaces, time constraints, lack of social support, and misconceptions about the role of exercise in diabetes management may further reinforce these barriers [14].

The findings also suggest that regular attendance at diabetes education sessions is significantly associated with a higher likelihood of PLWD's engagement in physical activity. The observed association resonates with that of a quasi‐experimental study in Iran [37] that found significant improvement in health beliefs and increased participation in physical activity after PLWD had been exposed to a diabetes education programme. A possible explanation is that such education sessions serve as a platform for enhancing participants' knowledge about the benefits of physical activity, clarifying misconceptions, and boosting confidence in their ability to incorporate exercise into their daily routines [37].

We also observed significant sex differences, with women being less likely to engage in physical activity than their male counterparts. This result is consistent with that of Whipple et al. [38] whose systematic review found higher physical activity among male PLWD compared to females. Notwithstanding this, our finding is inconsistent with that of Van Do et al. [39]. In the Ghanaian context, caregiving responsibilities, domestic workload, limited discretionary time and cultural expectations may constrain women's opportunities to engage in intentional physical activity, even when they remain active through household tasks.

It must be noted that while previous studies [16, 21] have found no significant association between waist‐to‐hip ratio (WHR) and physical activity among PLWD, we found an inverse association. That is, PLWD with a higher WHR were less likely to engage in physical activity. We argue that individuals with higher WHR may also experience heightened self‐consciousness or social stigma, and thus discourage physical activity engagement. It is also possible that those with higher WHR may feel less capable of performing exercise safely, leading to avoidance behaviour.

### 4.1 Implications for Policy and Practice

The inverse relationship between physical activity and perceived barriers points to the urgent need to address environmental, social and psychological obstacles that hinder physical engagement. This means prioritising investment in safe, accessible community exercise spaces and culturally appropriate public awareness campaigns that destigmatise physical activity. Clinically, diabetes care teams should screen for perceived barriers and provide individualised counselling to help patients overcome them. Given the positive association between regular attendance at diabetes education sessions and physical activity, clinicians and public health practitioners must continuously encourage PLWD to regularly attend these sessions. Where the PLWD cannot make it to the clinic, arrangements should be made to make this education available to them in their homes. Such education programmes must also emphasise the benefits that come with staying physically active as a PLWD.

### 4.2 Strengths and Limitations

There are some limitations which we acknowledge. First, due to the cross‐sectional design of this study, we are unable to establish any causal relationships. The findings only reflect associations. A longitudinal or experimental study design could help establish causality. Also, the self‐reported nature of the SDSCA measure means that there is the possibility of recall and social desirability bias. However, the SDSCA's restriction to recall in the past seven days reduces the recall bias to a large extent. Future studies may benefit from incorporating objective measures of physical activity, such as accelerometers or pedometers, alongside self‐reported data. Also, the study was limited to only persons living with T2DM; hence, the findings may not necessarily reflect the experiences of those living with type 1 diabetes. Nonetheless, the study contributes to expanding what is already known about physical activity among PLWD in low‐and‐middle‐income countries like Ghana. Also, the use of a backward stepwise approach in the regression modelling helped to eliminate redundancy and produce a more parsimonious model. Another limitation is that participants were recruited only from health facilities in the Ashanti Region of Ghana. Although one of the selected facilities is a major tertiary referral centre that serves several neighbouring regions, the sample may not fully represent all persons living with diabetes across Ghana. Regional differences in healthcare access, socio‐cultural practices, and opportunities for physical activity may limit the generalisability of the findings. Therefore, the results should be interpreted as context‐specific rather than nationally representative. Finally, the regression model explained a modest proportion of the variance in physical activity, indicating that other unmeasured factors, including environmental, social and structural determinants, may also influence engagement in physical activity among persons living with diabetes.

## 5 Conclusion

The study suggests moderate physical activity engagement among PLWD in Ghana. Perceived susceptibility, perceived benefits, and perceived barriers emerged as important cognitive determinants of physical activity, underscoring the relevance of health belief constructs in designing interventions. The findings suggest that diabetes management programmes should incorporate behavioural change models and prioritise targeted education and support, particularly for women and those in marital unions, to promote active lifestyles among PLWD in Ghana.

## Funding

The study received the Staff Development and Strategic Research Investment Funding from the School of Human and Health Sciences, University of Huddersfield, UK to support the data collection in Ghana (HHS013‐01). However, the content of this work is solely the responsibility of the authors and does not necessarily represent the official views of the University of Huddersfield.

## Ethics Statement

All methods in this study align with the Helsinki Declaration and Belmont Declaration. We obtained ethical clearance from the Ghana Health Service Ethics Review Committee (GHS‐ERC) [ID: GHS‐ERC: 004/02/23] and the School Research Integrity and Ethics Committee (SREIC) [ID: SREIC/2022/056] at the University of Huddersfield, UK. Written and oral informed consent was obtained from the participants.

## Consent

The authors have nothing to report.

## Conflicts of Interest

The authors declare no conflicts of interest.

## Data Availability

All relevant data are within the paper. Any other data or material associated with this manuscript are available on request through the first author (P.A.D.).
